# Complete Genome Sequence of a Carrot Torradovirus 1 Isolate, Obtained from *Angelica keiskei* in Japan

**DOI:** 10.1128/MRA.00110-19

**Published:** 2019-04-11

**Authors:** Ryosuke Tokuda, Masanobu Nishikawa, Naoi Hosoe, Takamichi Nijo, Nozomu Iwabuchi, Tetsuya Yoshida, Kiyoto Watanabe, Kensaku Maejima, Yasuyuki Yamaji, Shigetou Namba

**Affiliations:** a Department of Agricultural and Environmental Biology, Graduate School of Agricultural and Life Sciences, The University of Tokyo, Tokyo, Japan; Queens College

## Abstract

The complete genome sequence of the first Japanese isolate of carrot torradovirus 1 (CaTV1-J), which infects Angelica keiskei, was determined. This is the first report of a CaTV1 isolate obtained from *A. keiskei*.

## ANNOUNCEMENT

Carrot torradovirus 1 (CaTV1) is a member of the genus Torradovirus in the family *Secoviridae*; it possesses icosahedral particles and single-strand positive-sense bipartite RNA genomes designated RNA1 and RNA2 (1, 2). CaTV1 was first detected from carrots (Daucus carota, family Apiaceae) in the United Kingdom (1) and subsequently was detected in France (2). In addition to carrots, Torilis arvensis subsp. arvensis (family Apiaceae) was reported to be a natural host of CaTV1 in Greece (3). The complete nucleotide sequence of one CaTV1 isolate from the United Kingdom has been determined (GenBank accession numbers KF533719 and KF533720) (4). In this study, we detected a CaTV1 isolate from Angelica keiskei (family Apiaceae), a perennial herb used for food and medicine in Japan, and determined its complete genome.

In 2017, we collected A. keiskei leaves showing ring spot symptoms in Japan. Total RNA was extracted from the leaves using the ISOSPIN Plant RNA kit (Nippon Gene, Japan) and treated with DNase I (Nippon Gene). The cDNA library was constructed from the extracted RNA using the TruSeq RNA sample prep kit version 2 (Illumina, USA), according to the manufacturer’s instructions, except for poly(A)-tailed mRNA purification, and 2 × 100-bp paired-end sequencing was performed on a MiSeq instrument (Illumina) using the MiSeq reagent kit version 2 (500 cycles). A total of 1,859,186 paired-end reads were obtained, and adapter sequences and low-quality reads were removed using the Trimmomatic software version 0.36 (5) (ILLUMINACLIP:adapters.fa:2:30:10, LEADING:20, TRAILING:20 SLIDINGWINDOW:4:15, MINLEN:36, and other parameters at default settings). The reads were *de novo* assembled using the Trinity software version 2.5.1 (6), with default settings, and a total of 3,880 contigs (average read length, 392 bp; *N*_50_, 394 bp) were obtained. A BLASTx search (7) of the assembled contigs was performed against the GenBank database, and 20 contigs showing sequence identity with CaTV1 were obtained. The reads were mapped to the 20 contigs using the Bowtie 2 software version 2.3.4.3 (8), with default settings, resulting in an average coverage depth of 3.8×. The total length of the 20 contigs was 9,226 bp, covering 77% of the CaTV1 UK isolate genome, and 14 regions between contigs and the 5′- and 3′-terminal regions were undetermined. To determine the complete genome sequence of the Japanese CaTV1 isolate (CaTV1-J), undetermined regions between the contigs and 3′-terminal regions were amplified by reverse transcription-PCR using CaTV1-specific and oligo(dT) primer pairs, respectively. Amplified fragments were cloned into a pCR-Blunt II-TOPO vector (Invitrogen, USA), and 3 identical clones for each region were obtained. The 5′-terminal fragments were amplified using the 5′ rapid amplification of cDNA ends (RACE) system version 2.0 (Invitrogen) and cloned into the pCR2.1-TOPO vector (Invitrogen), and 2 identical clones were obtained.

The complete RNA1 and RNA2 sequences were 6,916 and 4,579 nucleotides (nt) long, respectively, excluding the poly(A) tails at their 3′ end. RNA1 encoded a 246-kDa polyprotein (nucleotides 147 to 6728), which contained the conserved motifs of a helicase (Hel), a protease (Pro), and an RNA-dependent RNA polymerase (Pol). RNA2 encoded a 22-kDa protein (nucleotides 197 to 787) of unknown function and a 130-kDa polyprotein (nucleotides 732 to 4229) predicted to be cleaved into a movement protein (MP) and three coat proteins (CPs). Sequence alignment with the CaTV1 UK isolate using the MUSCLE algorithm (9) in the program SDT version 1.2 (10) showed that the amino acid sequence identities of the Pro-Pol and CP regions of CaTV1-J with those of the UK isolate were 85 and 81%, respectively. Based on the current classification criteria for the family *Secoviridae* (11), CaTV1-J was found to be an isolate of CaTV1. To our knowledge, this is the first report of a CaTV1 isolate from *A. keiskei*.

### Data availability.

The genome sequence of CaTV1-J has been deposited in the DNA Data Bank of Japan/GenBank under accession numbers LC436363 and LC436364. The raw sequence data have been deposited in the National Center for Biotechnology Information Sequence Read Archive (SRA) under BioSample number SAMN11026531 and SRA run number SRR8640195, which are part of the SRA study number PRJNA524449.

## References

[1] Adams IP, Skelton A, Macarthur R, Hodges T, Hinds H, Flint L, Nath PD, Boonham N, Fox A 2014 *Carrot yellow leaf virus* is associated with carrot internal necrosis. PLoS One 9:e109125. doi:10.1371/journal.pone.0109125. 25365290 PMC4218861

[2] Rozado-Aguirre Z, Marais A, Svanella-Dumas L, Faure C, Latour F, Villeneuve F, Dickinson M, Fox A, Boonham N, Candresse T 2017 First report of *Carrot torradovirus 1* (CaTV1), a member of the *Torradovirus* genus, infecting carrots in France. Plant Dis 101:1333. doi:10.1094/PDIS-01-17-0095-PDN.

[3] Lotos L, Olmos A, Katis NI, Maliogka VI 2018 First report of *Carrot torrado virus 1* and *Carrot thin leaf virus* naturally infecting *Torilis arvensis* ssp. *arvensis* in Greece. Plant Dis 102:2049. doi:10.1094/PDIS-03-18-0381-PDN.

[4] Rozado-Aguirre Z, Adams I, Fox A, Dickinson M, Boonham N 2017 Complete sequence and genomic annotation of carrot torradovirus 1. Arch Virol 162:2815–2819. doi:10.1007/s00705-017-3410-5. 28526965

[5] Bolger AM, Lohse M, Usadel B 2014 Trimmomatic: a flexible trimmer for Illumina sequence data. Bioinformatics 30:2114–2120. doi:10.1093/bioinformatics/btu170. 24695404 PMC4103590

[6] Haas BJ, Papanicolaou A, Yassour M, Grabherr M, Blood PD, Bowden J, Couger MB, Eccles D, Li B, Lieber M, MacManes MD, Ott M, Orvis J, Pochet N, Strozzi F, Weeks N, Westerman R, William T, Dewey CN, Henschel R, LeDuc RD, Friedman N, Regev A 2013 *De novo* transcript sequence reconstruction from RNA-seq using the Trinity platform for reference generation and analysis. Nat Protoc 8:1494–1512. doi:10.1038/nprot.2013.084. 23845962 PMC3875132

[7] Altschul SF, Gish W, Miller W, Myers EW, Lipman DJ 1990 Basic local alignment search tool. J Mol Biol 215:403–410. doi:10.1016/S0022-2836(05)80360-2. 2231712

[8] Langmead B, Salzberg SL 2012 Fast gapped-read alignment with Bowtie 2. Nat Methods 9:357–359. doi:10.1038/nmeth.1923. 22388286 PMC3322381

[9] Edgar RC 2004 MUSCLE: multiple sequence alignment with high accuracy and high throughput. Nucleic Acids Res 32:1792–1797. doi:10.1093/nar/gkh340. 15034147 PMC390337

[10] Muhire BM, Varsani A, Martin DP 2014 SDT: a virus classification tool based on pairwise sequence alignment and identity calculation. PLoS One 9:e108277. doi:10.1371/journal.pone.0108277. 25259891 PMC4178126

[11] Thompson JR, Dasgupta I, Fuchs M, Iwanami T, Karasev AV, Petrzik K, Sanfaçon H, Tzanetakis I, van der Vlugt R, Wetzel T, Yoshikawa N, ICTV Report Consortium. 2017 ICTV virus taxonomy profile: *Secoviridae*. J Gen Virol 98:529–531. doi:10.1099/jgv.0.000779. 28452295 PMC5657025

